# In vivo Identification and Specificity assessment of mRNA markers of hypoxia in human and mouse tumors

**DOI:** 10.1186/1471-2407-11-63

**Published:** 2011-02-09

**Authors:** Morten Busk, Kasper Toustrup, Brita S Sørensen, Jan Alsner, Michael R Horsman, Steen Jakobsen, Jens Overgaard

**Affiliations:** 1 Department of Experimental Clinical Oncology, Aarhus University Hospital, Aarhus, Denmark; 2 PET centre, Aarhus University Hospital, Aarhus, Denmark

## Abstract

**Background:**

Tumor hypoxia is linked to poor prognosis, but identification and quantification of tissue hypoxia remains a challenge. The hypoxia-specificity of HIF-1α target genes in vivo has been questioned due to the confounding influence of other microenvironmental abnormalities known to affect gene expression (e.g., low pH). Here we describe a new technique that by exploiting intratumoral oxygenation heterogeneity allows us to identify and objectively rank the most robust mRNA hypoxia biomarkers.

**Methods:**

Mice carrying human (FaDu_dd_) or murine (SCCVII) tumors were injected with the PET hypoxia tracer FAZA. Four hours post-injection tumors were removed, frozen, and crushed into milligram-sized fragments, which were transferred individually to pre-weighed tubes containing RNAlater and then weighed. For each fragment radioactivity per tissue mass and expression patterns of selected mRNA biomarkers were analyzed and compared.

**Results:**

In both tumour models, fragmentation into pieces weighing 10 to 60 mg resulted in tissue fragments with highly variable relative content of hypoxic cells as evidenced by an up to 13-fold variation in FAZA radioactivity per mass of tissue. Linear regression analysis comparing FAZA retention with patterns of gene expression in individual tissue fragments revealed that CA9, GLUT1 and LOX mRNA levels were equally and strongly correlated to hypoxic extent in FaDu_dd_. The same link between hypoxia and gene expression profile was observed for CA9 and GLUT1, but not LOX, in SCCVII tumors. Apparent in vivo hypoxia-specificity for other putative molecular markers of tissue hypoxia was considerably weaker.

**Conclusions:**

The portrayed technique allows multiple pairwise measurements of mRNA transcript levels and extent of hypoxia in individual tumors at a smallest possible volumetric scale which (by limiting averaging effects inherent to whole-tumor analysis) strengthen the conclusiveness on true hypoxia-specificity of candidate genes while limiting the required number of tumors. Among tested genes, our study identified CA9, GLUT1 and possibly LOX as highly specific biomarkers of tumor hypoxia in vivo.

## Background

It is well known that tumor hypoxia limits the efficacy of anticancer treatment and promotes tumor progression [1,2]. But the demonstration and quantification of tissue hypoxia, which is a prerequisite for effective and adequate treatment, remains problematic especially in clinical settings. Several studies have demonstrated that direct measurement of physically dissolved oxygen with tissue-invasive polarographic oxygen electrodes is able to accurately separate patients into different risk categories [3,4]. However, most tumors are inaccessible to the electrode without prior surgery and the method is technically demanding which prevents its routine use in clinical settings [5,6]. Clinically, more attractive methods include non-invasive imaging of hypoxia-selective fluorinated nitroimidazoles developed for positron emission tomography (PET) and blood oxygenation level dependent (BOLD) MRI, but these techniques are expensive and have profound weaknesses (for a review on imaging techniques see [7]). Histopathological evaluation of needle tumor biopsies remains the gold standard for definitive diagnosis of many cancers. More thorough gene-expression analysis of the same tissue samples may provide indirect knowledge on the tumor microenvironment.

Since the discovery of the transcription factor hypoxia-inducible factor 1α (HIF-1α), which orchestrates cellular adaptation to hypoxia (for a recent review see reference [8]), a huge effort has been made to identify HIF-1α regulated genes that are useful as hypoxia biomarkers. Although the expression of HIF-1α and its target genes are tightly regulated by pO_2 _under certain well-defined in vitro conditions, the situation is more complex in vivo. In accordance, it has been shown that oxygen electrode data correlate poorly with the tissue expression of putative hypoxia responsive genes assessed in biopsies. In addition, studies examining the extent of spatial overlap between exogenous administered hypoxia probes (e.g., pimonidazole) and endogenous protein markers in tumor sections have produced conflicting results (for thorough reviews see references [9-11]). Whether this relates to the confounding influence of other microenvironmental parameters known to affect gene expression (e.g., low pH and low glucose), fluctuating tumor oxygenation, sampling errors caused by intratumoral heterogeneity or interlaboratory methodological variation is unclear. Further studies that assess the specificity and heterogeneity of distribution of putative endogenous markers of hypoxia in vivo are thus warranted. Here we describe a new technique that by exploiting intratumoral oxygenation heterogeneity allows us to identify and rank in vivo hypoxia-responsive mRNA biomarkers.

## Methods

Subcutaneous flank tumors, established by injection of murine SCCVII or human FaDu_dd _squamous cell carcinoma cells into C3H/HenTaC or NMRI-*nu*/*nu *female mice, respectively, were used for experiments. The SCCVII cell line was a gift from Dr. D. Siemann (University of Florida, Gainesville, Florida.) whereas FaDu_dd _was a gift from Dr. Michael Baumann (Department of Radiation Oncology, Carl Gustav Carus University Hospital and Medical Faculty, Dresden, Germany). The study was performed in accordance with the national legislation and approved by the Danish Animal Experiments Inspectorate (Permission number: 2005/561-1015).

When tumors reached a size of 500 to 1000 mm^3^, non-anaesthetized mice were injected with ~10 MBq of the PET hypoxia tracer ^18^F-fluoroazomycin arabinoside (FAZA) and returned to their cages. FAZA was allowed to distribute for 4 hours before tumors were removed; this time period ensures excellent hypoxia-specificity as shown in previous studies [12,13]. Subsequently, excised tumors were frozen. A piece of the thigh muscle was also collected to determine tracer background contamination (unbound tracer) in well-oxygenated tissue. Frozen tumors were placed in a dry-ice cooled aluminium box and crushed with a pair of pliers into milligram-sized fragments. Twelve to 20 appropriately sized fragments were collected from each tumor and transferred individually to pre-weighed tubes containing 100 μl of RNAlater solution (Qiagen), which prevents RNA degradation when samples thaw. Tubes were then weighed and radioactivity in each tumor fragment and a piece of weighed muscle was measured using a Packard well counter. Subsequently, tissue fragments were homogenized in 300 μl RLT buffer (Qiagen) containing 10 ml/l β-mercaptoethanol using a TissueLyser (Qiagen) and totRNA was extracted using the RNeasy mini kit (Qiagen) according to the manufactures instructions. Total RNA (2 μg) was reverse transcribed using random hexamer primers, and the High Capacity Archive kit (Applied Biosystems) according to the manufactures instructions. The relative levels of the following transcripts were measured by qPCR using Taqman Gene Expression assays (ABI): CA9 (human assay: Hs00154208_m1, mouse assay: Mm00519870_m1), GLUT1 (human assay: Custom Taqman Gene Expression assay, mouse assay: Mm01192270_m1), LOX (human assay: Hs00184700_m1, mouse assay: Mm00495386_m1 and Mm00495384_m1), LDHA (human assay: Hs00855332_g1, mouse assay: Mm00495282_g1), GAPDH: (human assay: Hs00266705_g1, mouse assay: Mm99999915_g1), ATF4 (human assay: Hs00909569_g1, Mouse assay: Mm00515324_m2). For each reaction cDNA (corresponding to 6 ng RNA), 1× assay mix and 1× Taqman Universal PCR mastermix (ABI) in a total of 15 μl was mixed. All reactions were performed in duplicate. Reactions were performed on an ABI Prism 7900 HT fast Real-Time PCR system (ABI). Results were normalised to three control genes: signal peptidase complex subunit 2 (SPCS2, human assay: Hs01585256_g1, mouse assay: Mm00651159_m1), ribosomal protein L37a (RPL37A, Human assay: forward primer TGT GGT TCC TGC ATG AAG ACA reverse primer GTG ACA GCG GAA GTG GTA TTG TAC probe 5TG GCT GGC GGT GCC TGG A, mouse assay: Mm01546394_s1) and Nedd4 family interacting protein 1 (NDFIP1, human assay: Hs00228968_m1, mouse assay Mm0128333_m1) which were selected on the basis of a previous microarray study [14], and the geNORM Visual Basic application available in RealTime Statminer (Intergromics). Data analysis was performed using the RealTime Statminer software (Intergromics), which calculates the geometric mean of the three control genes, and the Comparative C_T _method.

Finally, for each tumor fragment normalized mRNA transcript levels of individual genes were compared to relative FAZA retention (counts per weight of fragment/counts per weight of muscle) and results displayed as scatterplots on a per-tumor basis. The spatial link between gene expression and FAZA was analyzed using linear regression and Pearson's correlation coefficients.

## Results

**Figure 1:**
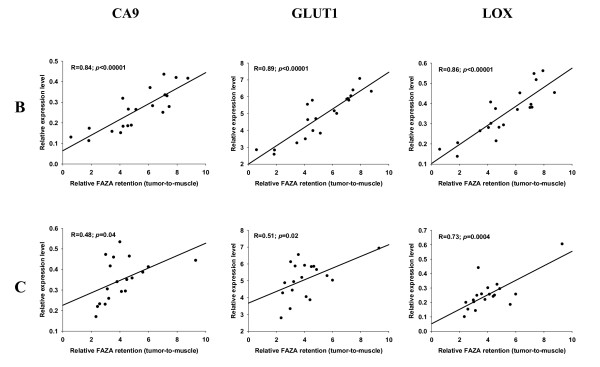
**Relative expression of mRNA levels as a function of FAZA retention**. Scatterplots showing the relationship between regional FAZA retention (relative to muscle) and expression levels of the three HIF-1α regulated genes CA9, GLUT1 and LOX in 2 representative fragmented FaDu_dd _xenograft tumors. Each dot represents matching values for a single tumor fragment.

**Figure 2:**
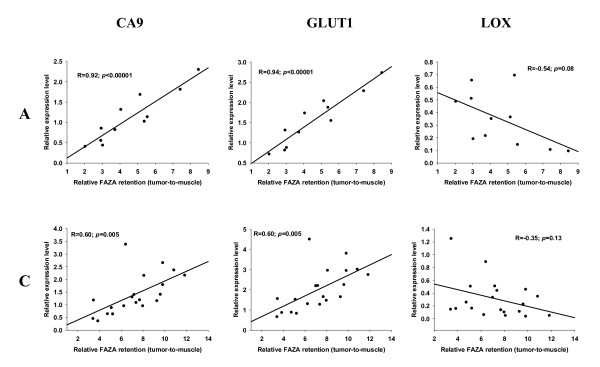
**Relative expression of mRNA levels as a function of FAZA retention**. Scatterplots showing the relationship between regional FAZA retention (relative to muscle) and expression levels of the three HIF-1α regulated genes CA9, GLUT1 and LOX in 2 representative fragmented murine SCCVII tumors. Each dot represents matching values for a single tumor fragment.

**Figure 3:**
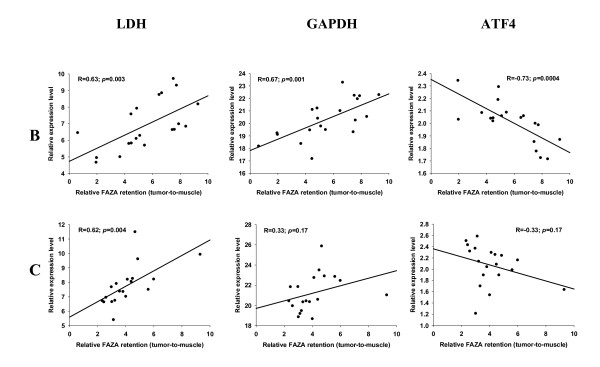
**Relative expression of mRNA levels as a function of FAZA retention**. Scatterplots showing the relationship between regional FAZA retention (relative to muscle) and expression levels of the two HIF-1α regulated genes LDH and GAPDH and the non-HIF-1α regulated gene ATF4 in 2 representative fragmented FaDu_dd _xenograft tumors. Each dot represents matching values for a single tumor fragment.

**Figure 4:**
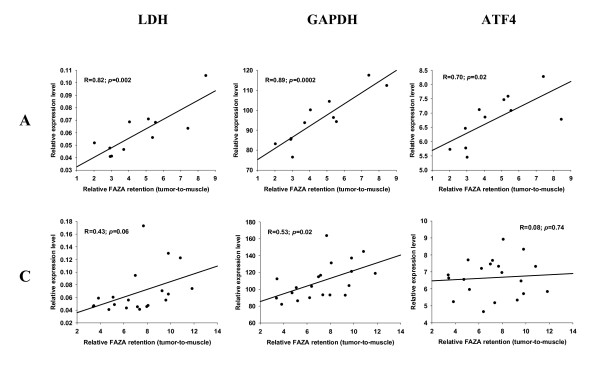
**Relative expression of mRNA levels as a function of FAZA retention**. Scatterplots showing the relationship between regional FAZA retention (relative to muscle) and expression levels of the two HIF-1α regulated genes LDH and GAPDH and the non-HIF-1α regulated gene ATF4 in 2 representative fragmented murine SCCVII tumors. Each dot represents matching values for a single tumor fragment.

A prerequisite for the usefulness of the technique portrayed in the present work is that the erratic tumor fragmentation results in fragments that differ widely in their relative content of hypoxia, as quantified by relative FAZA retention. This requirement was met since fragmentation into pieces weighing 10 to 60 mg resulted in an up to 13-fold and 6-fold variation in radioactivity per mass of tissue in FaDu_dd _and SCCVII tumors, respectively. Among a selection of well-known HIF-1α regulated genes CA9 and GLUT1 were highly significantly correlated to relative FAZA retention with nearly identical Pearson correlation coefficients (representative examples are given in Figure 1 and 2 and all results are summarized in Table 1), suggesting high hypoxia-specificity in vivo. LOX mRNA expression displayed a species difference with a strong positive correlation between fold-induction and hypoxic extent in the human tumor model (Figure 1 Table 1) and no or a weak inverse relationship in the murine tumor model (Figure 2 Table 1). Because of this unexpected tumor-type dependent difference in expression pattern of LOX, the results in SCCVII tumors were confirmed with an independent assay for LOX, which align in a different region of the transcript. The two assays generated remarkably similar results (additional file 1 figure S1). LDH transcript levels were also elevated in hypoxic tissue fragments (Figure 3 and 4, Table 1) but in the murine tumor model the spatial link was significantly weaker than for CA9 and GLUT1. GAPDH mRNA levels were positively and significantly correlated to FAZA retention in the murine tumor (Figure 4 Table 1), whereas no clear association was observed in the xenograft (Figure 3 Table 1). We also analyzed the link between FAZA and the transcription factor ATF4, which has previously been shown to be anoxia-induced in a HIF-1α-independent manner [15]. The linkage between ATF4 expression level and FAZA retention was weak to modest with substantial tumor-to-tumor variability, but the positive correlation in SCCVII tumors was significant in two out of four tumors and when all data were pooled (Figure 4 Table 1). In contrast a significant inverse correlation between relative ATF4 mRNA and FAZA was observed in three out of four FaDu_dd _tumors and when data were pooled (Figure 3 Table 1).

**Table 1:** Summary of Pearson regression coefficients (*R*) and *P *values when comparing relative gene expression and relative FAZA retention in small tumor fragments obtained from 4 human FaDu_dd _tumors and 4 murine SCCVII tumors.

FaDu_dd_	CA9		GLUT1		LDH		GAPDH		LOX		ATF4	
	*R*	*P*	*R*	*P*	*R*	*P*	*R*	*P*	*R*	*P*	*R*	*P*
A	0.77	≈10^-4^	0.85	3×10^-6^	0.72	< 10^-3^	0.17	0.48	0.77	< 10^-3^	-0.65	0.003
B	0.84	5×10^-6^	0.89	< 10^-6^	0.63	0.003	0.67	0.001	0.86	10^-6^	-0.73	< 10^-3^
C	0.48	0.04	0.51	0.02	0.62	0.004	0.33	0.17	0.73	< 10^-3^	-0.33	0.17
D	0.68	10^-3^	0.75	≈10^-3^	0.73	< 10^-3^	-0.1	0.69	0.75	< 10^-3^	-0.54	0.02
Mean	**0.69**		**0.75**		**0.67**		**0.18**		**0.78**		**-0.56**	

Pooled	0.58	< 10^-7^	0.74	< 10^-14^	0.52	< 10^-6^	0.13	0.24	0.76	< 10^-15^	-0.45	< 10^-4^

**SCCVII**	**CA9**		**GLUT1**		**LDH**		**GAPDH**		**LOX**		**ATF4**	
	***R***	***P***	***R***	***P***	***R***	***P***	***R***	***P***	***R***	***P***	***R***	***P***

A	0.92	< 10^-4^	0.94	< 10^-4^	0.82	0.002	0.89	< 10^-3^	-0.54	0.08	0.70	0.02
B	0.89	< 10^-6^	0.76	< 10^-3^	0.50	0.03	0.80	< 10^-4^	-0.48	0.03	0.49	0.04
C	0.60	0.005	0.60	0.005	0.43	0.06	0.53	0.02	-0.35	0.13	0.08	0.7
D	0.70	< 10^-3^	0.47	0.03	-0.10	0.67	0.16	0.51	-0.30	0.20	0.36	0.1
Mean	**0.78**		**0.69**		**0.41**		**0.59**		**-0.42**		**0.41**	

Pooled	0.65	< 10^-9^	0.64	< 10^-8^	0.24	0.04	0.56	< 10^-6^	-0.38	0.001	0.39	< 10^-3^

Analysis was performed on a per-tumor basis (A, B, C and D). Similar data analysis was also performed after data were pooled, i.e., for each gene the relative gene expression for all fragments for a given tumor type was plotted as function of relative FAZA content followed by regression analysis.

## Discussion

Pre- or during-treatment characterization of tumor hypoxia, which plays a major role in treatment failure and disease progression, may allow more effective individualized and modifiable therapy. However, due to methodological and interpretational challenges, inadequate facilities, or huge costs, several of the current available imaging techniques are still not in routine use. Since analysis of tumor biopsies is inexpensive and widely used in diagnosis, indirect assessment of the tumor microenvironment from genetic analysis in the same biopsies is clinically attractive if a set of reliable markers can be identified. Prognostic accuracy may be further increased by including other molecular markers with proven prognostic value (e.g., markers of energy metabolism, cell cycle regulation, metastatic potential, or stem cell characteristics).

Several studies have identified possible mRNA or protein hypoxia biomarkers based on in vitro cell culture studies, with special focus on genes that are under the control of HIF-1α [16]. However, the confounding influence of other tumor microenvironmental abnormalities like low glucose and/or low pH on HIF-1α target genes [17-20] or non-hypoxic HIF-1α activation by abnormal signalling or mutations [21,22] may be responsible for some of the spatial discrepancy observed between injectable hypoxia markers (e.g., pimonidazole) and O_2_-responsive gene protein products (reviewed in [9,10,23]). Accordingly, among the numerous candidate genes identified from cell culture experiments it is mandatory to identify those with a primarily hypoxia-driven gene expression (high specificity) and with high-fold hypoxic induction (high sensitivity) also in vivo.

Although proteins are the primary effectors of biological function, mRNAs are easier to detect and quantify, and thus more attractive as clinical biomarkers. In situ hybridization with RNA probes allows a spatial comparison between selected mRNA's and exogenous hypoxia probes [24,25]. However, the technique is not quantitative and therefore a clinically useful relationship between fold-induction of selected hypoxia-responsive genes and extent of hypoxia cannot be defined. In the present study we have developed a method that by comparing PET hypoxia tracer retention and gene expression profiles in small tumor fragments allows easy identification of in vivo hypoxia-responsive genes and objective ranking of these according to hypoxia-specificity. Multiple pair-wise measurements of gene expression profiles and hypoxia in individual tumors are superior to whole tumor analysis since the required number of tumors is reduced and, more importantly, volumetric downscaling increases conclusiveness on true hypoxia-specificity. Furthermore, variability of global-tumor hypoxic fraction will be reduced compared to variability in tumor fragments making it less likely to identify hypoxia-responsive genes in a study that relies on bulk tissue profiling of whole tumors. Fragmentation of tumors resulted in tissue fragments weighing from 10 to 60 mg with 4 to 6-fold differences in specific radioactivity in SCCVII tumors and up to 13 fold in FaDu_dd _tumors; much higher than the intertumor variability observed when assessing global tumor tracer retention in the same tumor models [12,26]. Indeed, the relatively weak correlations observed in the FADu_dd _tumor C (Figure 1) may be specifically linked to reduced fragment-to-fragment variability in relative FAZA content in that particular tumor. Fragmentation into even smaller tissue segments may, therefore, further increase the strength of this analysis, but the accuracy of the determination of the mass of individual fragments and the need for sufficient mRNA sets a lower size limit of ~5 mg tissue.

Our technique also depends on the ability of FAZA to accurately quantify relevant hypoxia. In general, a perfect match is not expected even between the induction of an ideal (i.e., strictly pO_2 _regulated) hypoxia biomarker and relative FAZA retention. First, K_m _(pO_2 _for half-maximal tracer binding/gene induction) for nitroimidazole-based compounds is lower than for HIF-1α regulated genes [18,19] and second, the induction levels of HIF-1α regulated genes plateaus below ~7.5 mmHg [18,19] whereas the uptake of nitroimidazoles increases dramatically when near-anoxic conditions are approached [27,28]. Nonetheless, such differences should not affect the ranking of genes according to hypoxia-specificity. In addition, it is well known that slow tracer distribution may compromise the quantitative accuracy of PET generated hypoxia-maps. Specifically, necrosis contains less tracer than viable well-oxygenated reference tissue (i.e., background) which weakens the correlation between mean tracer signal intensity and density of hypoxic cells in partly necrotic tumor regions [29]. However, in two previous studies comparing FAZA autoradiography and pimonidazole immunohistochemistry in a mouse tumor model we have shown that FAZA 3 to 4 h post-injection provides a highly accurate estimate of the regional number of hypoxic cells (pO_2 _< 10 mmHg) even in areas with hypoxic cells and necrosis intermixed [12,13]. This is linked to much more effective washout of unbound (contaminating) tracer at late time points in small animals than in patients, which maximize the tracer signal difference between hypoxic and well-oxygenated cells and minimize the difference between necrotic and well-oxygenated cells.

In contrast, and more importantly, in two tissue fragments with the same absolute number of hypoxic cells (and thus comparable FAZA signal) but variable content of necrotic tissue the number of hypoxic cells relative to the total number of viable cells will differ. Inclusion of such partly necrotic fragments would result in disproportionate high expression levels of hypoxia-responsive genes, which would appear as outliers above the regression line in the scattergrams. Inspection of regression lines suggest that this is not a significant problem, although the statistically identified outlier (residual >3 standard deviations above mean) in the murine tumor C (Figure 2) and another murine tumor (not graphed) may be linked to this problem. Removal of the single outlier in Figure 2 increases the regression coefficients substantially to 0.81 and 0.79 for CA9 and GLUT-1, respectively.

Transient changes in blood flow, which is common in solid tumors, may hide a real link between endogenous and exogenous hypoxia markers. Specifically, since oxygenation-driven increases or decreases in mRNA transcript levels may take hours (e.g., [19,20]) high levels of hypoxia-induced mRNA transcripts may still be present in recently reoxygenated areas (i.e., low FAZA retention) and newly developed tracer-retaining hypoxic areas may present with low levels of hypoxia-induced mRNA transcripts. Although the confounding factors discussed above may weaken a possible linkage between hypoxia and expression level of putative hypoxia-responsive genes, they are highly unlikely to affect the ranking of genes according to hypoxia-specificity or sensitivity.

Our study confirms that two classical markers of hypoxia, CA9 and GLUT1, also display high hypoxia-specificity in vivo in FaDu_dd _tumors as evidenced by highly significant correlations between local FAZA retention and relative gene induction in all tumors (Figure 1 Table 1). Also the expression of LOX which has been shown to play a crucial role in hypoxia-induced metastasis [30] was strongly linked to regional hypoxia in FaDu_dd _(Figure 1 Table 1). A highly significant association between LDH expression level and hypoxia was also observed, but for a given change in relative FAZA retention the change in LDH expression level (a reasonably estimate of sensitivity as opposed to specificity) is lower than for CA9, GLUT1 and LOX, making it less ideal as an in vivo hypoxia biomarker (compare Figure 1 and 3). Reduced in vivo sensitivity of LDH is in accordance with in vitro data from our laboratory showing that the hypoxia-induced change in induction of HIF-1α-regulated genes (i.e., hypoxia sensitivity) varies substantially, with the order of fold-induction as follows CA9≥LOX>GLUT1 > LDH in FaDu_dd _cells. The lack of difference in sensitivity between CA9, GLUT1 and LOX in the present study was not expected based on in vitro data, but may be linked to a differential influence of other deviant microenvironmental factors on HIF-1α regulated genes. For example, it has been shown that glucose-deprivation stimulates the expression of GLUT1 in myocytes and fibroblasts [31] and tumor cells [32] and that glucose deprivation and hypoxia has an additive effect on GLUT1 expression [33]. In contrast, the expression of CA9 protein (no available mRNA data) is prevented in HT1080 and FaDu tumor cells at 0.55 mM glucose [16]. Since glucose deprivation and hypoxia is likely to coexist, the apparent superiority of CA9 observed in vitro may not be present in vivo.

The relationship between relative CA9, GLUT1 and LDH expression and FAZA retention in SCCVII tumors was very similar to the human tumor xenograft. Intriguingly, however, in SCCVII tumors LOX expression was uncorrelated or even weakly inversely correlated to tracer retention (Figure 2 Table 1), possibly suggesting a fundamental species difference in the regulation of LOX. As this finding was unexpected, the validity of the results in the murine tumor was confirmed by a different LOX qPCR assay.

GAPDH has been used as a housekeeping reference gene in numerous studies. However, GAPDH is under the control of HIF-1α [34] and is part of the molecular manifestation of the Pasteur effect [35] and it is surprising that GAPDH has been extensively used as a reference gene in studies involving lowered pO_2 _[36,37]. In our hands, GAPDH has consistently been shown to be induced by hypoxia in vitro (unpublished), and Zhong and Simons [38] reached the same conclusion in a study on a diverse selection of tumor and non-transformed cell lines. In contrast, Said and colleagues [39] reported that GAPDH mRNA was insensitive to lowered pO_2 _in glioblastoma-derived tumor cells and these findings were later confirmed in other cell lines [40]. The reason for this fundamental inter-study discrepancy is unclear, but considering the number of cell lines tested methodological differences should be considered first. In the present study a weak, but in some tumors significant, correlation between FAZA retention and GAPDH expression was observed in both the human and murine tumor model (Figure 3 and 4, Table 1), suggesting that GAPDH is neither an appropriate reference gene nor an ideal hypoxia marker in vivo.

Several oxygen sensing pathways have been identified [41] and non-HIF regulated pO_2 _responsive genes may also be useful as hypoxia biomarkers, especially if the pO_2 _level of gene induction is lower than for HIF-1α target genes allowing a sub-population of severely hypoxic cells to be identified and quantified. ATF4, part of the unfolded protein response which is involved in the adaptation of cells to severe hypoxia/anoxia, was shown to be up-regulated by hypoxia at the level of mRNA in rat fibroblasts [15]. However, in the human breast cancer cell line MDA-MB 435 ATF4 mRNA levels were unaffected by pO_2 _whereas protein levels were highly elevated during near-anoxic conditions, strongly suggesting that regulation is mainly at the level of translational efficiency [42]. Our in vivo results revealed a weak, albeit in some tumors significant, positive (SCCVII, Figure 4 and Table 1) or negative (FaDu_dd_, Figure 3 and Table 1) correlation between ATF4 mRNA transcript levels and FAZA retention, suggesting that ATF4 is not a reliable non-HIF-1α regulated marker of severe hypoxia at the level of mRNA.

## Conclusions

We have developed a technique which allows a quantitative comparison of hypoxic extent and relative gene expression. A strong positive correlation between regional CA9, LOX and GLUT1 mRNA transcript levels and FAZA retention was revealed in a human tumor model suggesting that these genes display high hypoxia-specificity in vivo and are superior to LDH and GAPDH. Inclusion of several xenograft tumor models may allow us to identify robust in vivo hypoxia-specific tumor-type-independent biomarkers and construct clinically useful calibration curves that define the relationship between biopsy gene expression signatures and extent of hypoxia. The fragmentation technique may also be useful for establishing correlations between other labelled tracers and molecular changes (e.g., FLT and Ki-67, FDG and GLUT1/hexokinase).

The authors declare that they have no competing interests.

## Authors' contributions

MB participated in designing the study, carried out the experiments and grafted the manuscript. SJ produced the PET hypoxia tracer. KT, BBS, JA, MRH, SJ and JO participated in designing the study and the preparation of the manuscript. All authors read and approved the final manuscript.

## Pre-publication history

The pre-publication history for this paper can be accessed here:

http://www.biomedcentral.com/1471-2407/11/63/prepub

## Supplementary Material

Additional file 1 **Dct plot for two different LOX probes**. Dct values obtained from twenty different tumour fragments (Samples) from the two different LOX probes used (Mm00495386_m1 and Mm0095384_m). RPL37A, SPCS2 and Nedd4 are the used control genes. Click here for file
