# Supplementary figures and images for: In vivo Identification and Specificity assessment of mRNA markers of hypoxia in human and mouse tumors
Source: BMC Cancer. 2011 Feb 9;11:63. doi: 10.1186/1471-2407-11-63 (PMC3042974; doi:10.1186/1471-2407-11-63)

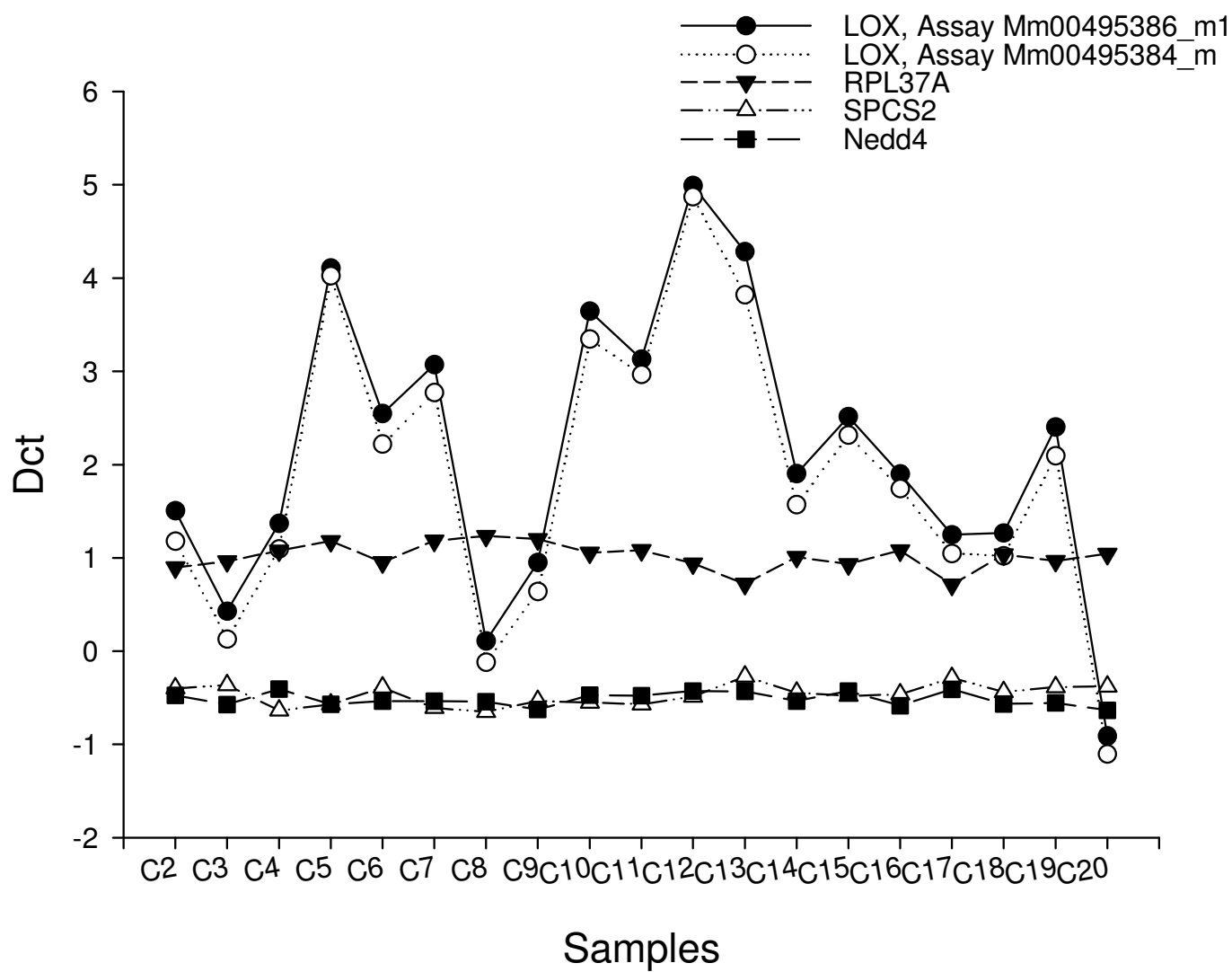

Supplementary figure 1

Supplement: Additional file 1 — Dct plot for two different LOX probes. Dct values obtained from twenty different tumour fragments (Samples) from the two different LOX probes used (Mm00495386_m1 and Mm0095384_m). RPL37A, SPCS2 and Nedd4 are the used control genes. [file 1471-2407-11-63-S1.PDF]
